# Estimating biodiversity changes in the Camargue wetlands: An expert knowledge approach

**DOI:** 10.1371/journal.pone.0224235

**Published:** 2019-10-24

**Authors:** Sara Fraixedas, Thomas Galewski, Sofia Ribeiro-Lopes, Jonathan Loh, Jacques Blondel, Hugo Fontès, Patrick Grillas, Philippe Lambret, Delphine Nicolas, Anthony Olivier, Ilse R. Geijzendorffer

**Affiliations:** 1 Tour du Valat, Research Institute for the conservation of Mediterranean Wetlands, Le Sambuc, Arles, France; 2 School of Anthropology and Conservation, University of Kent, Canterbury, United Kingdom; 3 Centre for Functional and Evolutionary Ecology–French National Centre for Scientific Research (CEFE-CNRS), UMR 5175, Montpellier, France; 4 French Odonatological Society (SfO), Bois d'Arcy, France; Universidad de Murcia, SPAIN

## Abstract

Mediterranean wetlands are critical strongholds for biodiversity and the provision of ecosystem functions and services; yet, they are being severely degraded by a number of socio-economic drivers and pressures, including climate change. Moreover, we still lack comprehensive understanding of the extent to which biodiversity loss in Mediterranean wetlands will accelerate change in ecosystem processes. Here, we evaluate how changes in biodiversity can alter the ecosystem of the Camargue (southern France). We collected data on species presence/absence, trends and abundance over a 40-year period by combining observations from the scholarly literature with insights derived from expert knowledge. In total, we gathered more than 1500 estimates of presence/absence, over 1400 estimates of species abundance, and about 1400 estimates of species trends for eight taxonomic groups, i.e. amphibians, reptiles, breeding birds, fish, mammals, dragonflies (odonates), orthopterans and vascular plants. Furthermore, we used information on recently arrived species and invasive species to identify compositional changes across multiple taxa. Complementing targeted literature searches with expert knowledge allowed filling important gaps regarding the status and trends of biodiversity in the Camargue. Species trend data revealed sharp population declines in amphibians, odonates and orthopterans, while birds and plants experienced an average increase in abundance between the 1970s and the 2010s. The general increasing trends of novel and invasive species is suggested as an explanation for the changing abundance of birds and plants. While the observed declines in certain taxa reflect the relative failure of the protection measures established in the Camargue, the increasing exposure to novel and invasive species reveal major changes in the community structure of the different taxonomic groups. This study is the first attempt to assess changes in biodiversity in the Camargue using an expert knowledge approach, and can help manage the uncertainties and complexities associated with rapid social-ecological change in other Mediterranean wetlands.

## Introduction

There is well-established evidence that Mediterranean wetlands are critical strongholds for biodiversity in the face of global change [1–4]. However, due to the increasing pressure from human activities such as drainage, pollution and agricultural intensification, Mediterranean wetlands are being severely degraded and biodiversity is being lost at alarming rates [5–8]. Moreover, given their crucial ecological importance at the global level and the wide array of ecosystem services that they provide from local to regional scales, safeguarding Mediterranean wetlands is critical to the implementation of the Sustainable Development Goals and the post-2020 global biodiversity framework [2,4].

The Camargue is the delta of the Rhône River and one of the largest and most biodiverse Mediterranean wetlands. Major changes in land-cover, land-use and water management have taken place over the last decades [8,9]. For instance, the area has experienced rapid agricultural intensification (including rice cultivation) and crop production during the last 50 years [10], including market gardening. The Camargue is an excellent example of the co-evolutionary dynamics of nature-culture interactions and a good case study of the state of biodiversity that is applicable to similar Mediterranean wetlands. In these habitats, the arrival of new species (some of them considered as invasive) and the extinction of native species have largely unknown consequences on species communities and ecosystem functioning [11–13,14].

Obtaining reliable estimates for changes in species richness and/or abundance over time is challenging due to a paucity of data and lack of baseline information for many species [15,16]. Moreover, several studies have shown that published observations are often biased in favour of species with a high cultural and/or conservation value [8]. For global indices, incomplete species datasets are often complemented with modelling techniques [17–20]. Yet, at regional- and/or local-scale, additional information on species richness and abundance are available through the knowledge and insights of experts who have worked in the area for a long time [21,22]. The time period for studying species population changes using expert knowledge typically spans a few decades, i.e. the time that the experts have worked in a particular area [23]. The use of experts may, therefore, allow species trends to be estimated over longer time periods and for a wider range of taxa than is possible using data based on field counts, especially when the use of long-term datasets is constrained due to for instance changes in sampling methods [24]. Although the use of expert estimations has its own limitations [25,26], it is one of the few methods available [27], and is used in the assessment of threatened species for the IUCN Red List [28]. Expert knowledge can complement observations from the available literature to piece together trends in regional biodiversity and provide more information on ecological baselines [29,30].

In this paper, we present a new and innovative approach to assess the state of the Camargue’s biodiversity. First, we evaluate the information gained from complementing species trends and abundance estimates available in the scholarly literature with expert knowledge estimates spanning over the last 40 years. Second, we use presence/absence and abundance estimates in relation to eight different taxa–amphibians, reptiles, breeding birds, fish, mammals, dragonflies (hereafter odonates), orthopterans and vascular plants–to determine the extent of ecological changes that have taken place from the 1970s up to the present time. Third, we test for differences in trends among taxonomic groups and assess whether these trends are influenced by recently arrived species (both non-native and invasive); we also test for the sensitivity of the trends to the confidence level given by experts. We finally discuss our findings on the drivers of biodiversity loss in the Camargue and reflect on the method used to monitor the status and trends of biodiversity in other Mediterranean wetlands where quantitative long-term datasets for most taxonomic groups are lacking.

## Material and methods

### Case study

**Fig 1:**
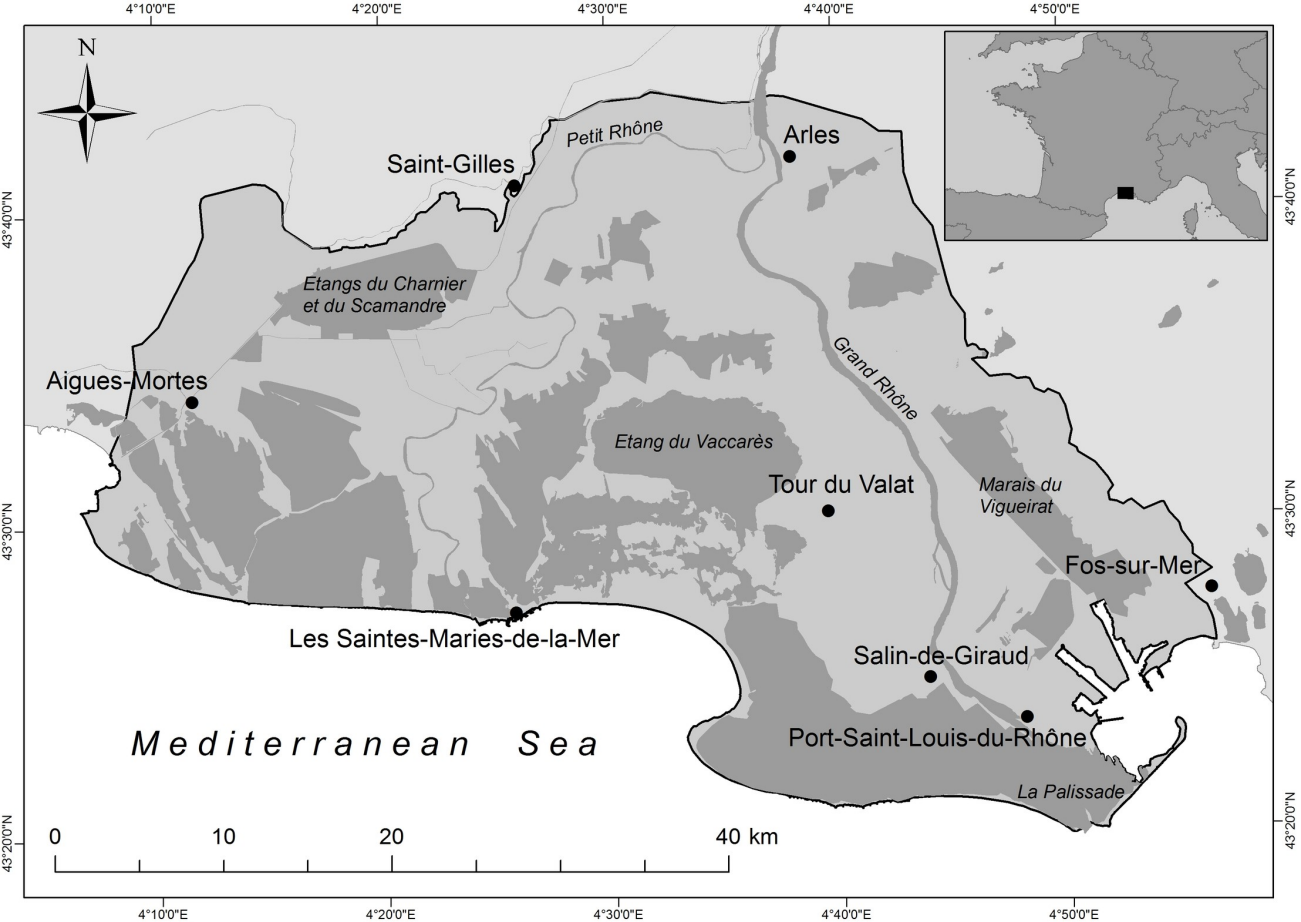
Study area of the Camargue (southern France). The study area is delimited by a black line. Light and dark grey indicate land and water, respectively. Note that the marine part of the area was not included.

This study focuses on the Camargue (Fig 1), one of the most biodiverse wetlands in the Mediterranean basin [3,31]. The study area comprises approximately 135,000 ha. It was designated as a Ramsar site in 1986 for its international importance for nesting, staging and wintering waterbirds [10,32].

We chose a time period of approximately 40 years starting from the 1970s because it coincides with historical data coverage in the scholarly literature for the majority of taxa. Given the number of ecological studies already existing for several taxonomic groups, it seemed reasonable to find experts with first-hand experience over this period. Furthermore, a large part of the Camargue was designated at that time as both a Biosphere Reserve (1977; covering a total of 193,000 ha) and a Natural Regional Park (1970; over 80,000 ha), with the aim of promoting the conservation of biodiversity as well as the traditional socio-economic activities in the area [10,33]. We selected taxonomic groups for which there were both data in the literature and experts to evaluate them. The chosen taxa were amphibians, reptiles, breeding birds, fish, mammals, odonates, orthopterans and vascular plants (more details in S1 Appendix).

### Expert consultation

The research design of this study is in accordance with the Ethical Review Board in the Humanities and Social and Behavioural Sciences of the University of Helsinki and followed the codes of ethics of the American Anthropological Association. All participants who were involved in this research were invited to be co-authors of this publication, with only those who showed an interest becoming part of the article’s authorship.

Before the start of the research, we obtained oral consent from all the participants involved in the study. We explained the project in detail to the participants, stressing that participation was strictly voluntary and that participants could opt out of the research at any point. We also emphasized that the information collected would be made available in the form of a scientific publication. Participation did not involve any cost to participants except for the time they gave during the evaluations based on the recruitment that took place at the Tour du Valat research institute and/or with online surveys. We also asked the participants whether they would like the information to be returned to them individually in any specific format. Data were collected on paper, and later digitized and stored in databases only accessible to the research team. At the end of the data collection (both workshops and online surveys), all information obtained was shared with participants at a dedicated workshop at the Tour du Valat (February 2018), where preliminary results of the project were presented.

During July and August 2017 we applied a snowball sampling technique [34] to find experts from different areas of the Camargue who would have in-depth expertise on the species in the study area during the selected period for each of the chosen taxonomic groups. The starting point for the snowball sampling technique were experts working at the Tour du Valat. From there, we identified and contacted other experts who have worked in the area, targeting those who had long-term experience observing species and the social-ecological changes in the study area. More than half of the experts initially contacted (n = 68) composed the final sample of experts (see Table 1).

**Table 1:** New data obtained from experts through workshops and online surveys, and existing data based on the literature consulted.

Taxonomic group	Date	Experts	Species considered	Species Pres/Abs	Species Abund	Species Trend	Species All
**New data from experts**							
Birds	22-08-17	9	132	132	132	132	132
Amphibians	08-09-17	4	10	9	9	10	9
Reptiles	08-09-17	4	16	16	16	16	16
Mammals	12-09-17	6	58	58	58	38	38
Plants	20-09-17	6	1263	1154	1152	1106	1101
Fish	27-09-17	8	54	52	52	54	52
Odonates	22-11-17	4	55	53	53	33	32
Orthopterans	22-11-17	4	84	51	1	13	1
**Total**		**44**	**1672**	**1525**	**1473**	**1402**	**1381**
**Existing data from literature**							
Birds			132	132	37	50	32
Mammals			58	58	57	13	13
**Total**			**190**	**190**	**94**	**63**	**45**

Dates when workshops took place, number of experts who participated in each consultation (either physically or via email), number of species initially considered and number of species for which we obtained at least one estimation (for presence/absence “Pres/Abs” and abundance “Abund” in both study periods, trends “Trend”, and for all presence/absence, abundance and trend metrics “All”). Note that some experts (n = 9) were able to participate in more than one evaluation (e.g. breeding birds and mammals), providing expertise for more than one taxon. Previous existing estimates for the species considered in this study were only found for birds and mammals from the literature search.

A total of six expert workshops were carried out at the Tour du Valat between August 2017 and November 2017 (Table 1) for the eight different taxa. Upon arrival, each expert was provided with a list including all the species for a given taxon and occurrence data (presence/absence values) obtained from the scholarly literature (more information in S1 Appendix). These records from the literature were obtained by reviewing documents held at the library of the Tour du Valat, the largest documentation centre on the natural history of the Camargue. These included several books and monographs on the social-ecological history of the Camargue (see references consulted in S1 Appendix). During the same workshops, experts were asked to verify and complement the species list and occurrence data for each species on the 1970s and the 2010s. We defined 1970 as roughly represented by species observations and relevant literature from the period 1965–1975, and 2010 from the period 2005–2015 (see details in S1 Appendix and S1 Table). Experts then categorized species trends between the 1970s and the 2010s as “increased”, “stable” or “declined”, and allocated each species to an abundance category for each period. The abundance categories were agreed upon by participants during the workshops and varied among taxa. For example, experts felt confident to allocate the abundance of bird species to one of six different categories on a logarithmic scale (0−1 to 10,000−100,000 individuals) whereas reptile and amphibian species were described using only three categories (“absent”, “rare” or “common”). To measure the confidence level of expert estimates, we asked them to provide a confidence score for each estimate (trend and abundance values) from 0 to 5 (details on the different categories defined for the trends, abundance and confidence scores can be found in S2 Appendix).

Although each expert initially evaluated each species independently, they were encouraged to discuss among themselves (e.g. a species that was rare and/or poorly documented) and/or consult literature when in doubt. Experts were free to disagree with existing literature if it did not correspond with their own personal observations. Experts that could not participate at the given date were offered the possibility to contribute via an online survey after the workshop was held (see more details about the online surveys in S3 Appendix).

Additionally, we carried out a validation analysis, crosschecking trend estimates obtained from experts with those derived from the literature consulted (see S2 Table).

### Changes in occurrence and abundance

Changes in species occurrence (presence/absence data) were calculated based on the number of species that appeared or disappeared from the study area (i.e. average of presence/absence values given by experts in the 1970s and the 2010s for each species in each taxonomic group).

Abundance scores were calculated for each species and each time period, and then weighted according to the degree of agreement among experts. Abundance confidence scores were weighted based on the degree of confidence given by experts (a detailed explanation can be found in S2 Appendix). For each taxonomic group, we used Welch Two Sample *t*-tests, which allow for heteroscedasticity, to find significant differences between the average weighted abundance and average weighted confidence scores in the 1970s and the recent time (i.e. changes in abundance and confidence scores, respectively). Note that differences in abundance and confidence scores could not be tested for orthopterans due to the limited number of estimations obtained from the experts.

### Modelling species trends

We used trend data to model species trends across all taxonomic groups (information equally coded; see S2 Appendix). We constructed a trend score (hereafter weighted trend) using the values assigned to trend categories (1 for increasing trends, 0 for stable trends, and –1 for declining trends) and calculated a weighted trend for each species in each taxonomic group following the same method as for abundance (see S2 Appendix for more information). The species-specific weighted trends were later on defined as the response variable in our models (see below). Using the information on confidence scores associated to trend estimates, weighted confidence scores were calculated for each species trend as done for abundance confidence scores (see S2 Appendix).

In addition, to explain potential taxonomic differences in trends and to detect changes at the ecosystem level, we considered two main components:

#### Novelness

Defining whether the species were new for the Camargue region (if they appeared during the 2010s) or native (if they were already present in the 1970s) based on the occurrence data (average of presence/absence values among all experts who evaluated a particular species through workshops and/or online surveys). Experts were asked to corroborate that the identified number of new arriving species was realistic and not the result of observation effort biases [35] or taxonomic changes (especially in the case of plants, since differences in species taxonomic classification occurred between the 1970s and the recent time). Only in less than 1% of all species trend estimates, the presence or absence of a species could not be corroborated for a certain period due to lack of information from both literature and experts.

#### Invasiveness

Defining whether species were considered as invasive or non-invasive regardless of their classification as native or novel species; see definition of *biological invasion* [36]. This information was obtained from the European list of alien invasive species [37] and later on reviewed and improved by experts from the Tour du Valat who adapted the list to the Camargue. The Camargue list of invasive fish [38] was complemented with data from the National Museum of Natural History in France (MNHN). The plant list of invasive species was complemented with the publication of Terrin et al. [39].

These two components were then integrated in a single variable called “nov-inv”, in which each species was classified in one of the four categories: “0” for non-novel and non-invasive species, “1” for non-novel and invasive species, “2” for novel and non-invasive species and “3” for novel and invasive species (see S1 Fig for a distribution of the species in each category).

In order to evaluate which subset of explanatory variables best described patterns in species trends, we made use of information-theoretic model selection [40]. The set of explanatory variables used to construct the competing candidate models included:

- taxa-ID: identity of the taxonomic groups, set as a factor variable.

- nov-inv: variable defining both the novelness and invasiveness component of each species, set as a factor variable.

- CS: weighted confidence score values, set as a continuous variable, calculated using the information on confidence scores associated with species trend estimates (see above). We included this covariate to check whether trend values were correlated with confidence scores (e.g. lower confidence scores being associated to more negative trend values).

Because the novelness and invasiveness component was assumed to differ between taxonomic groups, we included interactions between “nov-inv” and “taxa-ID”. The competing models had all the possible subsets of explanatory variables whose maximum Pearson’s correlation coefficients were below 0.5 to avoid problems with collinearity [41]. We finally obtained a total of 10 different model combinations that were evaluated according to their parsimony based on their AIC (Akaike’s Information Criterion) [40] values and assuming normally distributed residuals (see Table 2). In all models, we used the previously calculated species-specific weighted trends as the response variable.

**Table 2:** The 10 candidate models explaining patterns in species trends evaluated based on their AIC values.

**Model**	***k***	**Δ**_**i**_	***w***_**i**_
**Trend ~ taxa-ID + nov-inv**	**11**	**0.00**	**0.547**
**Trend ~ taxa-ID + nov-inv + CS**	**12**	**0.39**	**0.451**
Trend ~ taxa-ID * nov-inv	32	12.92	0.001
Trend ~ taxa-ID * nov-inv + CS	33	12.96	0.001
Trend ~ nov-inv + CS	5	58.86	9e-14
Trend ~ nov-inv	4	59.61	6e-14
Trend ~ taxa-ID + CS	9	447.77	3e-98
Trend ~ taxa-ID	2	449.65	1e-98
Trend ~ 1	1	498.77	3e-109
Trend ~ CS	2	499.02	2e-109

*k* is the number of explanatory variables, Δ_i_ the AIC differences compared to the most parsimonious model, and *w*_i_ the AIC weights. The model indicated as “Trend ~ 1” includes only the intercept. In the models containing the taxonomic identity of the groups (“taxa-ID”), birds were selected as the reference group (intercept). Similarly, in the models containing the variable “nov-inv”, non-novel and non-invasive species were also set as the intercept. CS is the weighted confidence score. The most parsimonious models are in bold.

We fitted multiple linear regression models to each of the 10 candidate models using the “lm” function in R version 3.4.3 [42]. *F*-tests were calculated to test the significance of the predictors in the models. Differences in species trends between the different categories of the variable “nov-inv” (factor with 4 levels) were tested using pairwise multiple comparisons.

## Results

### Complementing literature with expert knowledge data

Our final dataset consisted of 1525 species estimates of presence/absence (91% of the total number of species initially considered), 1473 species abundance estimates (88%), and 1402 species trend estimates (84%; Table 1). For 1381 species all three metrics (species presence/absence, abundance and trends) were estimated (83%; Table 1).

Almost all trend and abundance estimates obtained through expert consultation were new additions (i.e. species evaluated for the first time for which there were not previous estimates in terms of trends and/or abundance in the literature consulted). We collected a total of 1339 new species trend estimates (96% from all species trend estimates) and 1379 abundance estimates (94% from all species abundance estimates). Even for breeding birds and mammals, for which a considerable number of estimates of trends and/or abundance for the selected period were already available in literature (Table 1), we were able to obtain new information for more than 50% of the bird species found in the Camargue in terms of trends (62%) and also in terms of abundance (72%). Trend estimates from experts matched in 90% of cases with those obtained from literature for birds and in 92% of cases for mammals. Only in the case of birds there was one species whose trends from experts were found to be the opposite compared to literature (S2 Table).

In general terms, we obtained a good proportion of data for the three metrics considered. The main exception were orthopterans, with experts only being able to identify the presence/absence of 61% of the 84 species initially considered and the abundance of one species for both study periods (Table 1). Therefore, this is the taxonomic group with the least information available, and only 15% of the 84 identified species were evaluated in terms of trends. More information on trends can be found in S3 and S5 Tables and S4 Appendix.

### Occurrence and abundance changes

**Fig 2:**
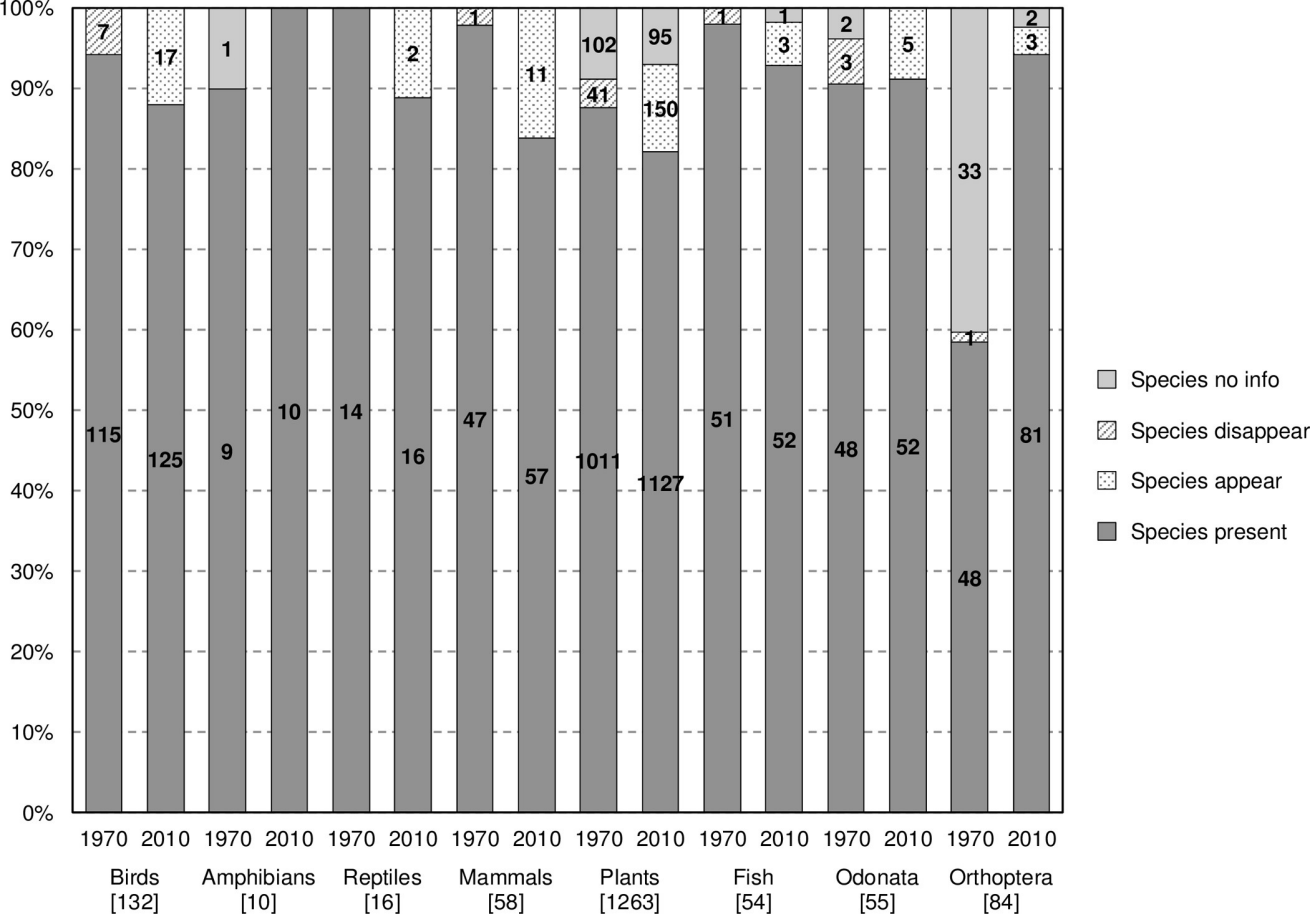
Species occurrence changes in the Camargue based on the average of presence/absence values given by experts. Dark grey columns depict the number of species present in the Camargue in the 1970s and the 2010s for each taxonomic group. The graph also shows the number of species that appeared after the 1970s, the number of species that disappeared from the study area, and number of species with no information on their occurrence. Categories are represented as percentages in order to be compared between taxa. Numbers in brackets indicate the species initially considered during the expert evaluations.

From all species initially evaluated (n = 1672), a total of 191 species (11%) were identified as “new arrivals” (i.e. species that were not present in the Camargue in the 1970s) based on presence/absence data. Of the taxa, mammals had most new species arriving (19% of the species list per taxa; Fig 2). The total number of species that disappeared from the 1970s to the 2010s was 54 (3%). Birds and odonates were the two taxonomic groups with most species disappearing (5% of the species list per taxa in both cases; Fig 2).

When comparing the average abundance for each taxonomic group between the 1970s and the 2010s, we found a significant increase for breeding birds (Welch Two Sample *t*-test: m_1970s_ = 2.4, m_2010s_ = 2.7, *df* = 255.94, *t* = 2.03, *p* = 0.043) and vascular plants (Welch Two Sample *t*-test: m_1970s_ = 1.9, m_2010s_ = 2.1, *df* = 2280.10, *t* = 3.89, *p* < 0.001). This result suggests that birds and plants have become more abundant during the last 40 years, with birds having experienced an average change of one category of abundance (more information in S4 and S5 Tables). We were not able to identify any significant change in abundance for other taxonomic groups. Across taxa, experts consistently tended to be more confident about estimates for recent observations in comparison to estimates for the 1970s (see S4 Appendix).

### Trend model

Results from the information-theoretic approach revealed that there were two models supported over the others in terms of parsimony (AIC weights of the first and second best models: *w*_i_ = 0.547 and *w*_i_ = 0.451, respectively; difference in AIC between the best and the third best model was Δ_i_ > 12.92; Table 2). Both best models included the predictor “taxa-ID” (taxonomic identity of the group), which had significant effects on species’ trends (best model: *F* = 10.71, *df* = 7, *p* < 0.001; Table 3).

**Table 3:** Coefficients and test values of variables explaining the patterns in trends shown only for the most parsimonious model.

Variable	Estimate	SE	*t*-value	*p*-value
Best model:	Trend ~ taxa-ID + nov-inv			
Intercept	**−0.138**	**0.053**	2.59	0.010
Plants	0.052	0.056	0.93	0.355
Amphibians	**−0.585**	**0.209**	2.79	0.005
Reptiles	*−0*.*272*	*0*.*161*	1.69	0.091
Mammals	*−0*.*185*	*0*.*112*	1.65	0.100
Fish	−0.058	0.100	0.59	0.558
Odonates	**−0.585**	**0.120**	4.88	< 0.001
Orthopterans	**−0.862**	**0.191**	4.52	< 0.001
Non-novel invasive	**0.527**	**0.086**	6.15	< 0.001
Novel non-invasive	**1.056**	**0.057**	18.42	< 0.001
Novel invasive	**1.080**	**0.083**	12.94	< 0.001
Residual SD	0.608	−	−	−
Adjusted R^2^	0.296	−	−	−

The estimated residual standard deviation (Residual SD) and adjusted R^2^ are also presented in the table. Statistically significant (*p*-value < 0.05) coefficients are in bold and tendencies (*p*-value < 0.1) are italicised. Note that breeding birds (“taxa-ID”) and non-novel non-invasive species (“nov-inv”) are defined as the intercept in the model.

**Fig 3:**
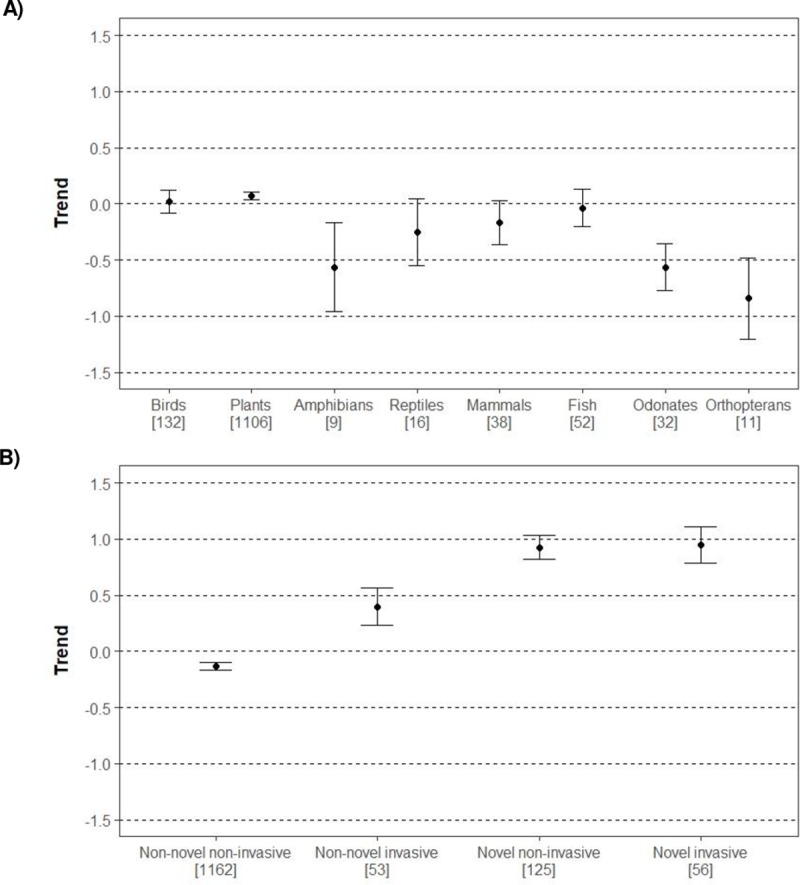
Predicted trends and 95% confidence intervals from the best model given: A) identity of the taxonomic group (“taxa-ID”), and B) novelness and invasiveness component of each species (“nov-inv”). Note that trends are predicted based on 1396 observations because six species could not be classified in any of the four represented categories in B). Trend values range from −1 (decline) to 1 (increase) according to categorization of trends made by experts. See Methods section for a description of the variables included in the best model (Table 3).

Trends of orthopterans, odonates and amphibians were significantly declining compared to birds, the reference group. In addition, trends of reptiles and mammals were almost significantly differing from birds (Table 3 and Fig 3).

Both best models also included the predictor “nov-inv” describing the novelness and invasiveness characteristics of the species. This variable had also significant effects on species trends (best model: *F* = 165.00, *df* = 3, *p* < 0.001; Table 3). Trends of non-novel invasive species, novel non-invasive species and novel invasive species were significantly increasing as compared to non-novel non-invasive species, the reference group (Table 3 and Fig 3). In addition, non-novel (native) invasive species had significantly lower trends as compared to both novel non-invasive (Pairwise comparison: *b* = –0.529 ± 0.100 SE, *p <* 0.001) and novel invasive species (Pairwise comparison: *b* = –0.553 ± 0.117 SE, *p <* 0.001). The second best model included the weighted confidence scores “CS” calculated from the information on confidence scores given by experts, but this variable had no influence on species trends (*b* = 0.160 ± 0.127 SE, *df* = 1384, *t* = 1.27, *p =* 0.206).

## Discussion

The expert workshops and posterior online surveys allowed an enriched understanding of the status and trends of biodiversity in the Camargue. Apart from changes in occurrence of species in the system (i.e. presence/absence), the most important data obtained refer to the identification of changes in species’ abundances from the 1970s to the 2010s. An analysis by taxonomic group cannot reveal why such changes happened unless most species within a taxonomic group share similar life-history traits and/or habitat preferences. This is partially the case for amphibians and odonates, which are closely linked to freshwater wetlands, and orthopterans, where many species are associated with grasslands. Based on the results from the trend model, amphibians, odonates and orthopterans were the three taxa showing the most significant declining trends. Temporary ponds and grasslands reduced in surface area by around 60% between 1942 and 1984 [43]. These two habitats have declined the most in the Camargue, having been converted into farmland or industrial areas. Therefore, it is likely that the severe degradation of the conservation status of these three taxonomic groups is related to the loss of those habitats. The modification of hydraulic management [44] has contributed significantly to the decline of amphibians and odonates over the past forty years. In addition to changes in hydrology [45], the use of selective insecticides to control mosquito larvae has been shown to decrease the abundance and species richness of odonates [46]. Other factors have had an impact, such as the massive contamination of aquatic environments by pollutants of agricultural and industrial origin [47]. The change in agricultural practices over the last decades has contributed to the disappearance of the threatened *Sympetrum depressiusculum* [48,49], which was very abundant in the rice fields of the Camargue a few decades ago [50]. Finally, the colonization of the Camargue by the exotic red swamp crayfish *Procambarus clarkii* has had an impact on odonates [51], and probably on some amphibian species such as the palmate newt *Lissotriton helveticus*, although no impact could be demonstrated on the Mediterranean tree frog *Hyla meridionalis* [52]. Although orthopterans were identified as the group showing the starkest declines, so far no study has investigated the drivers of these trends either in the Camargue or in other Mediterranean wetlands. However, experts consistently agreed during the workshop that this group suffered from the use of herbicides and insecticides, e.g. *Chorthippus* spp. [53].

Within other taxonomic groups considered in this study, species are found in a wider diversity of habitats and it is harder to relate changes in species abundance or richness to changes in ecosystem extent or functions. In each of these groups we find increasing, declining and stable species. Both mammals and reptiles showed almost significant declining trends. The intensification of agricultural practices and the loss of natural habitats might explain this tendency. Although mammals were the group with most species naturally arriving in the Camargue, e.g. small carnivorous species such as the common genet *Genetta genetta*, the beech marten *Martes foina* or the pine marten *Martes martes* [54–57], populations of many mammalian species have declined sharply in the last decades, probably due to changes in agricultural practices [56,57]. However, one of the most spectacular and dramatic declines, that of the European rabbit *Oryctolagus cuniculus*, is attributed to the appearance of new epizootic diseases [58]. For the ocellated lizard *Timon lepidus*, the disappearance of rabbit warrens has driven the species to the brink of extinction due to the lack of suitable sites [59]. Similarly, the significant increase in wild boar *Sus scrofa* populations in the delta [56] is having a strong impact on reptiles [60].

While vascular plants did not show any noticeable change in trends, our results revealed that this group experienced a significant increase in average abundance between the 1970s and the present time. This pattern might be explained by the introduction of novel species, including highly invasive species (e.g. water primrose *Ludwigia* spp., groundsel tree *Baccharis halimifolia* and several others). These species may have benefitted from the construction of irrigation and drainage canals, increasing plant productivity in wetlands and developing woody ecosystems along canals, and the increased nutrient loads in arable lands [44,61–63]. This contrasts with the decrease of some plant populations, especially those of temporary ponds and dry grasslands [44,64].

Birds became more abundant during the study period. Several authors argued that this group may have benefitted from different conservation actions and management schemes as part of the implementation of the EU Birds and Habitat Directives [7,65]. Additionally, changes in water management regimes may have facilitated the colonization and/or niche expansion by new bird species, including several waterbirds and raptors that formerly suffered from uncontrolled hunting and human persecution in the Camargue, e.g. several species of herons and egrets [66,67]. Also, while some species may have suffered from the arrival of invasive species (e.g. amphibians, odonates), for others this has meant an increase in food resources and in consequence a remarkable boost in their populations, e.g. some bird species [68]. Although some newly arrived bird species strongly increased in the Camargue (including the common wood pigeon *Columba palumbus* or the great egret *Ardea alba*), birds were one of the groups with most species disappearing from the study area in the last 40 years. Farmland birds in particular have declined since the 1950s [8]. However, because of the significant differences in confidence scores between the 1970s and the 2010s (i.e. lower confidence in our ecological baseline), changes in abundance for breeding birds and vascular plants must be regarded with caution.

Overall, our study provides evidence that the biodiversity of the Camargue ecosystem has deteriorated, with amphibians, odonates and orthopterans showing some of the sharpest declines. The patterns of increases and declines in different species groups reflect major changes in the compositional structure of the studied taxa, and indicate that the Camargue has undergone significant changes with important implications for local ecosystem functioning. These changes have taken place despite the different protection measures established in the Camargue since the 1970s (i.e. the Natural Regional Park and the Biosphere Reserve) and other protection measures already in place, e.g. the Camargue National Reserve, created in 1927 [33]. While these measures have clearly proved to be efficient for several bird species, they have failed to preserve the overall species diversity. We may therefore be unaware of the loss of biodiversity in other sites in the Mediterranean basin where protection measures are lacking and less expert knowledge is available.

Our results were limited by expert knowledge and the availability of scholarly literature over the study period, which determined the taxa and temporal coverage [69]. Despite differences in experts judgements, and sometimes limitations on data collection (e.g. decline of certain common species overlooked by experts), the method delivered robust outcomes about different taxa, even for species groups such as invertebrates, for which there are currently little or no data. Odonates were one of the groups with the largest number of species disappearing, and almost all orthopteran species that were considered to be declining according to experts have been classified as threatened in the Mediterranean region [70]. Obtaining estimates for these groups is particularly relevant to determine the extent of biodiversity loss. Although data from literature was only available for birds and mammals, the high percentage of matches in trend estimates between expert knowledge and published studies for these two groups provides an indication of the expertise and quality of the information contributed by the participants.

Acknowledging that major land-use changes have already taken place in the study area between late 1960s and early 1970s, e.g. channelization of the Rhône river, embankment [71,72], which are probably not fully captured in our data, we were able to detect some interesting patterns of change in a period of only 40 years through the use of expert judgement. Moreover, our results align well with those obtained from the computation of the Living Planet Index of Mediterranean wetlands [7,73] and the Living Region Index of the Provence-Alpes-Côte d’Azur [74], but we further refine them by broadening the temporal resolution and incorporating some of the previously neglected taxa. However, more research is needed to provide a more comprehensive understanding of the responses of the Camargue ecosystem to particular drivers of biodiversity loss (e.g. pesticide use, wetland management) and how they affect species assemblages.

This study represents the first attempt to monitor changes in biodiversity in the Camargue using an expert knowledge approach. We have demonstrated the value of complementing scholarly literature with expert knowledge, which opens new avenues for data collection, notably for identifying trends. Considering the urgent need to reverse biodiversity loss in Mediterranean wetlands, and taking into account the lack of long-term datasets for most taxonomic groups, our method could be used as a complementary tool to monitor the status and trends of biodiversity in data-deficient regions, and help manage the uncertainties and complexities associated with rapid social-ecological change.

## Supporting information

S1 Appendix Content of tables and compilation of information used in workshops. Content of the tables used in expert workshops and subsequent online surveys, and compilation of information used for species evaluation for each taxonomic group. Note that, as a general rule, we did not consider certain species in the survey if there was a common consensus that they were absent from the Camargue in both study periods.
(DOCX)

S2 Appendix Categorization of metrics and computation of the weighted trends, abundances and confidence scores. Trend, abundance and confidence score categories defined for each of the included taxa, and calculations made to obtain the weighted trends, abundances and confidence scores. Note that experts were not always able to provide trend and abundance estimates for all species, and only estimates associated to a certain confidence score were considered for the computation of the weighted trends and abundances.
(DOCX)

S3 Appendix Details of online surveys. Additional information from the online surveys.
(DOCX)

S4 Appendix Averaged weighted confidence scores for trends and abundance. Results from averaged weighted confidence scores for trends and abundance.
(DOCX)

S1 Table Example of tables used in workshops and online surveys. Example of the content of surveys (in this case for amphibians, in French and English) used during the workshops and online surveys. The content shown in this table was very similar as for the rest of the studied taxonomic groups, the only difference being the species evaluated and the type of qualitative information included. Class, family, scientific and common name of the species, qualitative information and presence/absence data for the two study periods obtained from literature were added to the table. Note that for some species, information on the presence/absence could not be determined before the workshop even with the previous consultation with an expert due to lack of background information and/or knowledge of the species at a particular time period (especially in the 1970s). Experts were asked to provide information on species trends and abundances (for both study periods) as well as confidence scores (CS in English, SC in French; see S2 Appendix) for more details). An additional space was left for comments from experts on a particular species.
(DOCX)

S2 Table Verification of raw trend data obtained from experts with species trend estimates derived from literature for both breeding birds and mammals. Trend estimates from experts were compared to trends derived from quantitative data, only known for species counted in all or almost the entire delta. Species with one asterisk refer to one category of discrepancy among trends (e.g. stable vs increase), whereas two asterisks indicate a two-level discrepancy among trends (i.e. opposite trends; decline vs increase).
(DOCX)

S3 Table Results from raw trend data. Number and percentage of increasing, declining and stable species for each of the taxonomic groups evaluated based on the average reply from experts (raw trend data). Categorization of trends was the same for all taxonomic groups (see S2 Appendix)). The highest percentages of declining species were identified for orthopterans, odonates and amphibians, while the highest percentage of increasing species belonged to mammals, plants and birds. The highest percentages of stable species were associated to fish, followed by birds and plants. For half of the taxonomic groups, the percentage of stable species was a bit higher than for increasing and declining species.
(DOCX)

S4 Table Results from raw abundance data. Number of species distributed in the different abundance categories for each taxonomic group and period (the 1970s and the 2010s) based on the average answer from experts (raw abundance data). Categorization of abundance was the same for amphibians, reptiles, mammals, fish, odonates and orthopterans (see S2 Appendix). For birds, numbers in brackets refer to individuals.
(DOCX)

S5 Table Average weighted trends, abundances and confidence scores. Average weighted species trends, abundances, distributions (except for birds and vascular plants) and confidence scores for each taxonomic group. See S2 Appendix) for more information on the different categories and given values. Note that only one orthopteran species was evaluated in the 1970s.
(DOCX)

S1 Fig Distribution of species in categories of variable “nov-inv”. Number of species classified in each of the four categories of the variable “nov-inv”: non-novel non-invasive species, non-novel invasive species, novel non-invasive species and novel invasive species for each taxonomic group. Species for which we could not confirm whether they were considered as new arrivals are also plotted. Note that numbers have been obtained from the trend database (n = 1402 species). Categories are represented as percentages in order to be compared between taxa. The majority of species were classified as non-novel non-invasive (83% from total). Amphibians and odonates had 90% or higher number of species belonging to this category. Novel non-invasive species were the second most popular group (9% from total). Mammals had most species belonging to categories non-novel invasive (8%), novel non-invasive (13%) and novel invasive (11%).
(TIF)
