# Supplementary material for: Estimating biodiversity changes in the Camargue wetlands: An expert knowledge approach
Source: PLoS One. 2019 Oct 24;14(10):e0224235. doi: 10.1371/journal.pone.0224235 (PMC6812746; doi:10.1371/journal.pone.0224235)
Supplement: S1 Appendix — Content of the tables used in expert workshops and subsequent online surveys, and compilation of information used for species evaluation for each taxonomic group. Note that, as a general rule, we did not consider certain species in the survey if there was a common consensus that they were absent from the Camargue in both study periods. (DOCX) [file pone.0224235.s001.docx]

Prior to each workshop, a table was prefilled with data from literature and the information was subsequently checked for errors by at least one expert of each of the included taxa. The prefilled table consisted of: a) a list of all species for a particular taxon thought to have an established population in the Camargue (i.e. several observations recorded), excluding all vagrant and strictly marine species (the latter being however frequent in the continental aquatic habitats of the Camargue); b) information on the presence (1) and absence (0) for each of the species in the past (the 1970s) and recent (the 2010s) times; and c) some information (usually qualitative) related to each species (for one or both of the study periods) intended to help the experts when providing their estimates (see S1 Table for an example). We also excluded exotic species that did not have an established population in France (e.g. the spur-thighed tortoise *Testudo graeca* or the pin-tailed whydah *Vidua macroura*) with the help of an expert.

Breeding birds

Earlier studies had shown that the breeding bird community of the Rhône Delta had suffered profound long-term changes in terms of structure and composition, and that these changes were coinciding with some important episodes of habitat loss [1]. To expand on previous research, breeding birds were selected over migrating and wintering species. In order to obtain the list of breeding bird species in the Camargue for our two study periods, we first consulted the list of breeding birds reconstructed by Galewski and Devictor [1]. We then used the bird guide written by Blondel and Isenmann [2] to extract information on the presence/absence of the species in the past (1965–1975) based on qualitative information about the species reproduction. For the present period (2005–2015), a recent ornithological report published by Kayser et al. [3] was employed to determine the species presence/absence together with the advice of a bird expert at the Tour du Valat. Absent species were usually defined as those having only one case of reproduction, not breeding at all in the Camargue during that particular period or simply when no breeding records were found. Present species were defined as having at least two cases of reproduction for a certain period. Based on these principles, we selected the species for which there were at least two cases of reproduction for one of the two time periods and excluded the rest of species for which reproduction only happened once, did not happened or was not confirmed/known. A combination of qualitative and quantitative information about the species in the past was collected from Blondel and Isenmaan [2] whereas the report by Kayser et al. [3] was used to look for information in recent times. Data included for instance information on the evolution of the species population in a certain period, how abundant and frequent was the species as a breeder, the approximate number of breeding couples or sites where the species was observed during the study period, or the main breeding habitat preferences of the species. The latter description was useful for experts who were able to link species’ populations development with changes occurred at the habitat level. For evaluating the species, some experts made us of the bird guide from Blondel and Isenmann [2] and other reports containing bird estimates for the study area.

Amphibians and reptiles

The species list was compiled using the doctoral thesis published by Guillaume [4], the atlas from Geniez and Cheylan [5], and the encyclopaedia of Blondel et al. [6]. Most of these sources were used to also extract information on species presence/absence based on abundance maps, which was complemented by an expert at the Tour du Valat. Whenever possible, additional information on the abundance, distribution or nativeness of the species (native meaning a species already present in the 1970s and novel otherwise) was added to the survey. For evaluating the species, some of the experts used the thesis of Guillaume [4] as inspiration.

Mammals

Most of the information included in the survey was obtained from the book of Poitevin et al. [7]. This book is the first to provide an overview of the history and evolution of mammals in the Camargue through texts describing, for each species, their biology and their past and present status, among other data, all complemented by original distribution maps. Similarly as for amphibians and reptiles, qualitative information such as abundance, distribution, nativeness and evolution of species populations was added to the survey. In addition, an article published by Kayser et al. [8] was used to complement the information for bat species. Some experts consulted casual observations that gave an indication of the change in species status.

Plants

Data were extracted using the Information and Location of Species System called SILENE (*Système d’Information et Localisation des Espèces*). At the Bouches-du-Rhône and Gard departments (including the Camargue), all plant observations are recorded and verified by the National Mediterranean Botanical Conservatory (*Conservatoire Botanique National Méditerranéen*). The initial list of plant species was made by extracting the plant observations from the perimeter of the Camargue Biosphere Reserve, whose limits resemble very much the ones of our study area. From this extraction, we used the number of plant observations (each observation only refers to one single point in time and place) in the 1970s and the present time, and add them to the plant survey to be used as a reference by experts. This information was also needed to obtain the presence/absence data. Although the old observations from SILENE are defined as “before the 1990s”, we used that information as a reference point for selecting the species. All genera and plant varieties were excluded from the main list, but complementary taxonomic classifications such as subdivisions of the genus into a group of species (*gpe*; when taxonomy was not clear or identical throughout the study period), hybrids and subspecies were kept. All species defined as “gpe” (n = 8) or subspecies (n = 133) were considered as single species following expert advice (e.g. averaging the abundance values obtained for each of the subspecies belonging to the same species was not done given the potential differences in abundance between subspecies). In most cases, experts made use of SILENE for evaluating the occurrence data and defining the trend and the abundance of each of the species. Whenever they found discrepancies in the observations or no data in SILENE, especially for the 1970s, experts consulted the inventory from Molinier and Tallon [9]. This inventory is regarded as a good knowledge synthesis about the abundance and distribution of plants in the Camargue before 1974. In addition, experts also consulted the catalogue from Molinier [10].

Fish

The list of species was compiled based on the work published by Crivelli [11], the identification guides from Keith et al. [12] and Louisy [13], and also the number of species found in the Fumemorte drainage canal and the Étang de Vaccarès lagoon (both located in the study area). In the sampling sites of Fumemorte and Vaccarès, fish communities have been monitored since the 1990s to present (Fumemorte) and up to 2008 (Vaccarès) by the Tour du Valat and the Camargue National Reserve (*Réserve Naturelle Nationale de Camargue*), respectively. Some of these sources were also used to obtain information on species presence/absence based on the catch rates (CPUE). Surveys included information on whether the species were classified as native or novel as well as their habitat type (e.g. fresh water, brackish water) or life cycle (migratory species). Qualitative information was obtained from Crivelli [11] and Rosecchi et al. [14] and was complemented by experts at the Tour du Valat. Whenever possible, additional information on the abundance, distribution or evolution of the species population was added to the survey using the same sources as for the rest of qualitative data. The evaluation of species by experts was generally done based on their expertise on a particular monitoring site (e.g. Vaccarès) within the study area.

Odonates and orthopterans

To compile the species lists for odonates and orthopterans, we consulted the management plan of the Tour du Valat Regional Nature Reserve written by Cohez et al. [15] and the MSc thesis about odonates and orthopterans by Merlet [16]. This gave us information on species presence/absence and their status (e.g. abundance, distribution, nativeness). For odonates, we also looked for quantitative data (number of individuals) from the National Odonata data collection programme (*Complément à l’inventaire des libellules de France*) known as « CILIF » (French Odonatological Society, *Société française d'odonatologie* – SfO) for the period 2014-2016 to help complementing the species list, as well as the doctoral thesis from Aguesse [17] and the revision of the species red list for the Provence-Alpes-Côte d’Azur region by Lambret et al. [18]. As for orthopterans, the list was further elaborated with the doctoral thesis of Bigot [19], one of Bigot’s works [20], and the red list for the Mediterranean region from Sardet and Defaut [21]. Both lists were checked and supplemented by an expert from the Tour du Valat. Lack of literature for the 1970s made difficult to obtain qualitative information for both groups (also for many orthopteran species we could not find information for their present status). However, for some species qualitative information on the species abundance and distribution could be added to the surveys.

**References**

1. Galewski T, Devictor V. When common birds became rare: Historical records shed light on long-term responses of bird communities to global change in the largest wetland of France. PLoS One. 2016; 11(11): e0165542.
2. Blondel J, Bourlière F, Isenmann P. Guide des oiseaux de Camargue. Delachaux & Niestlé; 1981.
3. Kayser Y, Blanchon T, Galewski T, Gauthier-Clerc M, Poulin B, Thibault M, et al. Compte-rendu ornithologique Camargue-Crau-Alpilles pour les années 2007-2012. Tour du Valat & SNPN / Réserve Nationale de Camargue; 2014.
4. Guillaume CP. Reptiles et batraciens de grande Camargue. Approche comparative avec la faune des marismas (sud-ouest de l'Espagne). Doctoral dissertation, Université de Montpellier. 1975.
5. Geniez P, Cheylan M. Les amphibiens et les reptiles du Languedoc-Roussillon et régions limitrophes. Atlas biogéographique. Mèze and Paris: Biotope & MNHN; 2012.
6. Blondel J, Barruol G, Vianet R. L’Encyclopédie de la Camargue. 1st ed. Paris: Buchet-Chastel; 2013.
7. Poitevin F, Olivier A, Bayle P, Scher O. Mammifères de Camargue. 1st ed. Castelnau-le-Lez: Regard du Vivant & Parc Naturel Régional de Camargue; 2010.
8. Kayser Y, Bayle P, Chambouleyron M, Disca T, Haquart A, Olivier A. Les chauves-souris de Camargue. Synthèse et actualisation des données. Le Vespère. 2009; 1: 36-57.
9. Molinier R, Tallon G. Documents pour un inventaire des plantes vasculaires de la Camargue. Bull Mus Hist Natur Marseille. 1974; 34: 7-165.
10. Molinier R. Catalogue des plantes vasculaires des Bouches-du-Rhône. Bull Mus Hist Natur Marseille. 1980; Tome IL.
11. Crivelli AJ. Les peuplements de poissons de la Camargue. Rev Ecol (Terre et Vie). 1981; 35(4): 617-671.
12. Keith P, Persat H, Feunteun É, Allardi J. Les poissons d’eau douce de France. Mèze and Paris: Biotope & MNHN; 2011.
13. Louisy P. Guide d’identification des poissons marins – Europe et Méditerrané. Ulmer; 2015.
14. Rosecchi E, Poizat G, Crivelli AJ. Introductions de poissons d’eau douce et d’écrevisses en Camargue : historique, origines et modifications des peuplements. Bull Fr Pêche Piscic. 1997; 344/345: 221-232.
15. Cohez D, Chauvelon P, Yavercovski N, Ernoul L. Plan de gestion 2011-2015 Réserve Naturelle Régionale Tour du Valat. Annexes. Tour du Valat; 2011.
16. Merlet F. Étude des peuplements d’odonates et d’orthoptères des principaux milieux à enjeux de la RNR de la Tour du Valat (Arles – 13). MSc thesis, Université de Montpellier. 2011.
17. Aguesse P. Contribution à l'étude écologique des zygoptères de Camargue. Doctoral dissertation, Université de Paris. 1961.
18. Lambret P, Ronne C, Bence S, Blanchon Y, Blettery J, Durand É, et al. Révision de la Liste rouge des libellules (Odonata) de Provence-Alpes-Côte d’Azur – version 2017. Martinia. 2017; 33: 37-52.
19. Bigot L. Essai d’écologie quantitative sur les invertébrés de la sansouire camarguaise. Doctoral dissertation, Aix-Marseille Université. 1961.
20. Bigot L. Ecologie des milieux terrestres salés. Bulletin de la Société d’écologie. 1971; 2: 99-121.
21. Sardet E, Defaut B. Les Orthoptères menacés en France. Liste rouge nationale et listes rouges par domaines biogéographiques. Matériaux Orthoptériques et Entomocénotiques. 2004; 9: 125-137.
