# Supplementary material for: Estimating biodiversity changes in the Camargue wetlands: An expert knowledge approach
Source: PLoS One. 2019 Oct 24;14(10):e0224235. doi: 10.1371/journal.pone.0224235 (PMC6812746; doi:10.1371/journal.pone.0224235)
Supplement: S2 Appendix — Trend, abundance and confidence score categories defined for each of the included taxa, and calculations made to obtain the weighted trends, abundances and confidence scores. Note that experts were not always able to provide trend and abundance estimates for all species, and only estimates associated to a certain confidence score were considered for the computation of the weighted trends and abundances. (DOCX) [file pone.0224235.s002.docx]

Trend and confidence score categories were the same for all taxa. Trends were defined based on three different categories.

↑ Increase

≈ Stable

↓ Decline

To calculate the weighted trend scores (more information below), each trend category was given a value. Increasing trends were defined as 1, stable trends as 0 and declining trends as –1.

Confidence scores were defined using six different categories on a scale ranging from 0 to 5. The meaning of each of the confidence scores was the following:

0 I do not know

1 I am uncertain

2 I doubt

3 I would say (I buy you a drink just in case)

4 I think (I bet a bottle of good wine)

5 I am sure (I bet my house)

Therefore, confidence scores ranging from 0 to 1 can be considered as low, 2–3 as medium and 4–5 as high confidence scores.

Contrary to trends and confidence scores, abundance categories differed among taxa. These abundance categories also referred to species distribution in the case of reptiles, amphibians, mammals, fish, odonates and orthopterans. In order to compute the weighted abundances, each category was given a value, as done with trends (see below).

Breeding birds

The abundance for birds was defined using six different categories, each of them representing a certain range of individuals (numbers in brackets).

A [0–1]

B [1–10]

C [10–100]

D [100–1,000]

E [1,000–10,000]

F [10,000–100,000]

These categories were given a value following an ordinal scale, where numbers ranged from 0 to 5.

Plants

The abundance of plants was also defined using six different categories, three of them describing the level of rarity and the rest referring to common species.

A Absent

B Very rare

C Rare

D Less frequent

E Common

F Very common

Similarly as for birds, abundance categories were given a value ranging from 0 to 5.

Amphibians, reptiles, mammals, fish, odonates and orthopterans

For these groups we used rougher categories to define both the abundance and distribution patterns of the species.

A Absent

RL Rare and localized

RW Rare but widespread

CL Common but localized

CW Common and widespread

To give a value to each of the categories, we split them into abundance (A = 0, R = 1, C = 2) and distribution (A = 0, L = 1, W = 2), since they refer to two different dimensions in which species rarity can be categorised [1,2].

To calculate the weighted abundance scores for each species and each time period, we first counted how many times experts “voted” for a certain abundance category. Each of these summed votes was then multiplied by the category value (see above) and divided by the total number of votes given by experts. For example, if three out of four experts said a certain species in the 1970s was “absent” one said “rare” and none said “common”, then the weighted abundance for this period would be (3 × 0 + 1 × 1 + 0 × 2) / 4 = 0.25. Using the information on confidence scores associated with abundance estimates for each study period (ranging from 0 to 5), a weighted confidence score was calculated for each species separately for the 1970s and the 2010s. Weighted confidence scores were estimated by dividing the total sum of “confidence score points” given to each abundance category by the maximum confidence score that the species could get based on the number of experts participating in the evaluation (if four experts were present, then this value would be 5 × 4 = 20). Following the previous example, if the four experts would have attributed a total of 13 points to category “absent” and 2 points to category “rare”, then the weighted confidence score associated to the abundance in the 1970s for that species would be 15 / 20 = 0.75. For those taxonomic groups in which experts provided information on species abundance and distribution simultaneously, weighted abundances were calculated separately from distribution related data. Weighted confidence score values were calculated the same way for all groups.

To calculate the weighted trend and confidence scores for each species, the same procedure was followed as for abundance.

**References**

1. Rabinowitz DS. Seven forms of rarity. In: Synge H, editor. The Biological Aspects of Rare Plant Conservation. Chichester: Wiley; 1981. pp 205-217.

2. Fattorini S, Sciotti A, Tratzi P, Di Giulio A. Species distribution, ecology, abundance, body size and phylogeny originate interrelated rarity patterns at regional scale. J Zool Syst Evol Res[. 2013; 51(4): 279-286.](https://lib.ugent.be/en/catalog/ejn01:954925573929)
