# Supplementary material for: Estimating biodiversity changes in the Camargue wetlands: An expert knowledge approach
Source: PLoS One. 2019 Oct 24;14(10):e0224235. doi: 10.1371/journal.pone.0224235 (PMC6812746; doi:10.1371/journal.pone.0224235)
Supplement: S3 Appendix — Additional information from the online surveys. (DOCX) [file pone.0224235.s003.docx]

We complemented the information obtained during the workshops by sending an online survey to either relevant experts who could not be physically present during the evaluations at the Tour du Valat or experts that did participate in a workshop but wanted to have more time to work on the table.

To provide all experts with the same starting point as well as to increase the return rate for the online surveys, the survey came with a guideline protocol containing definitions of trend, abundance and confidence scores, a description of the different categories to be used, some examples and a map of the study area. Presence/absence values derived from experts during the workshops were not modified in the online questionnaires unless everybody agreed about a certain species being present or absent from the system for any of the two study periods. However, because we needed to update the number of species present in the list almost after each workshop, we modified the original questionnaire and added presence/absence data for the new species based on the average answer obtained during the workshops.

In cases where, due to time constraints, experts could not complete the whole survey (particularly for birds and plants), they were contacted two weeks after the workshop (except for bird experts, who were contacted a month later after evaluating the first workshop). In those cases, we provided the experts with a personalized online questionnaire where we marked the species already evaluated, including information on the different trend, abundance and confidence score categories. Experts filled in the online survey for as many species as possible.

The complementary surveys took place from October 2017 until February 2018.
