# Supplementary material for: Estimating biodiversity changes in the Camargue wetlands: An expert knowledge approach
Source: PLoS One. 2019 Oct 24;14(10):e0224235. doi: 10.1371/journal.pone.0224235 (PMC6812746; doi:10.1371/journal.pone.0224235)
Supplement: S4 Appendix — Results from averaged weighted confidence scores for trends and abundance. (DOCX) [file pone.0224235.s004.docx]

Weighted confidence scores associated to trends of plants and orthopterans were the lowest among all included taxa. These are the taxonomic groups for which there is less information available regarding the species past and present status, and therefore the confidence score of trends given by experts was lower than for the rest of the groups. Conversely, the highest weighted confidence scores were given to trends of amphibians and reptiles (for more information, see S5 Table).

As for abundance, fish had among the highest weighted confidence scores in both the 1970s and 2010s. Reptiles had the highest confidence score in the 1970s and odonates in the 2010s. Significant differences between past and present weighted confidence scores (i.e. experts being more confident in the present) were found for birds (Welch Two Sample *t*-test: m_1970s_ = 0.17, m_2010s_ = 0.29, *df* = 251.91, *t* = 10.30, *p* < 0.001), plants (Welch Two Sample *t*-test: m_1970s_ = 0.17, m_2010s_ = 0.19, *df* = 2323, *t* = 3.43, *p* < 0.001), mammals (Welch Two Sample *t*-test: m_1970s_ = 0.27, m_2010s_ = 0.37, *df* = 103.54, *t* = 4.53, *p* < 0.001), fish (Welch Two Sample *t*-test: m_1970s_ = 0.49, m_2010s_ = 0.63, *df* = 101.79, *t* = 3.40, *p* < 0.001) and odonates (Welch Two Sample *t*-test: m_1970s_ = 0.25, m_2010s_ = 0.82, *df* = 54.22, *t* = 27.40, *p* < 0.001). This difference was almost significant for reptiles (Welch Two Sample *t*-test: m_1970s_ = 0.51, m_2010s_ = 0.61, *df* = 29.11, *t* = 1.90, *p* = 0.067). The fact that experts were most times more confident in the present than in the past could be explained by fewer number of experts with experience in the past as compared to the present (see also S5 Table).
