# Supplementary figures and images for: Estimating biodiversity changes in the Camargue wetlands: An expert knowledge approach
Source: PLoS One. 2019 Oct 24;14(10):e0224235. doi: 10.1371/journal.pone.0224235 (PMC6812746; doi:10.1371/journal.pone.0224235)

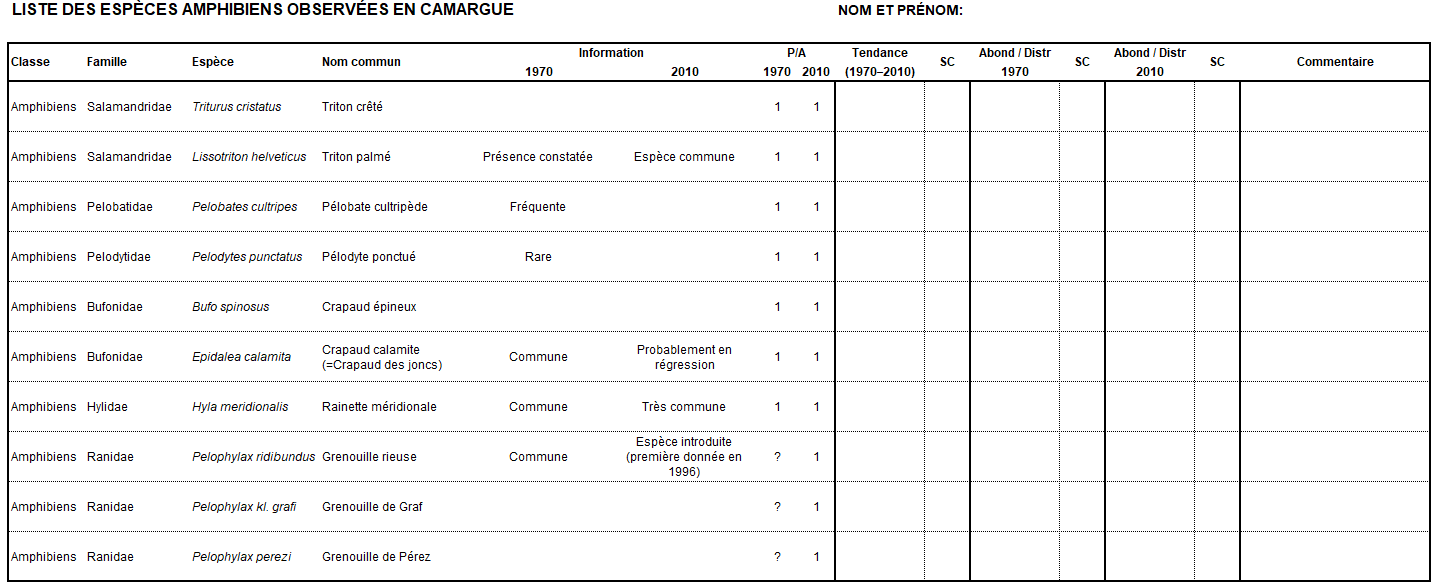


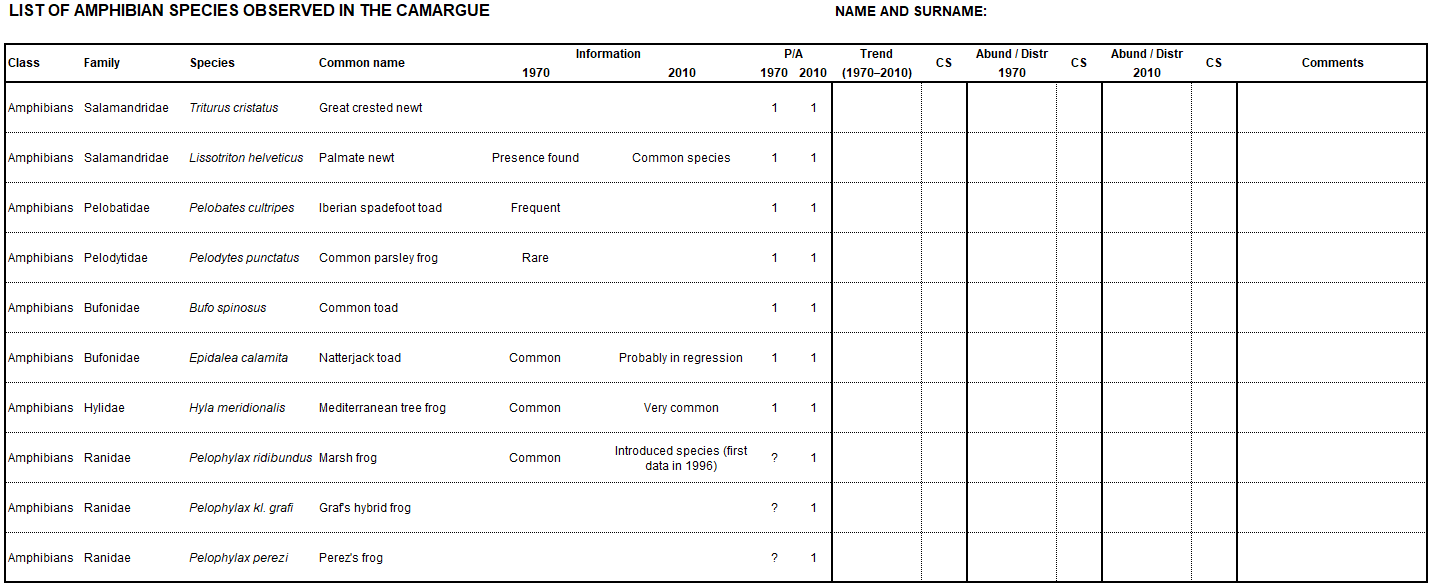

Supplement: S1 Table — Example of the content of surveys (in this case for amphibians, in French and English) used during the workshops and online surveys. The content shown in this table was very similar as for the rest of the studied taxonomic groups, the only difference being the species evaluated and the type of qualitative information included. Class, family, scientific and common name of the species, qualitative information and presence/absence data for the two study periods obtained from literature were added to the table. Note that for some species, information on the presence/absence could not be determined before the workshop even with the previous consultation with an expert due to lack of background information and/or knowledge of the species at a particular time period (especially in the 1970s). Experts were asked to provide information on species trends and abundances (for both study periods) as well as confidence scores (CS in English, SC in French; see S2 Appendix) for more details). An additional space was left for comments from experts on a particular species. (DOCX) [file pone.0224235.s005.docx]

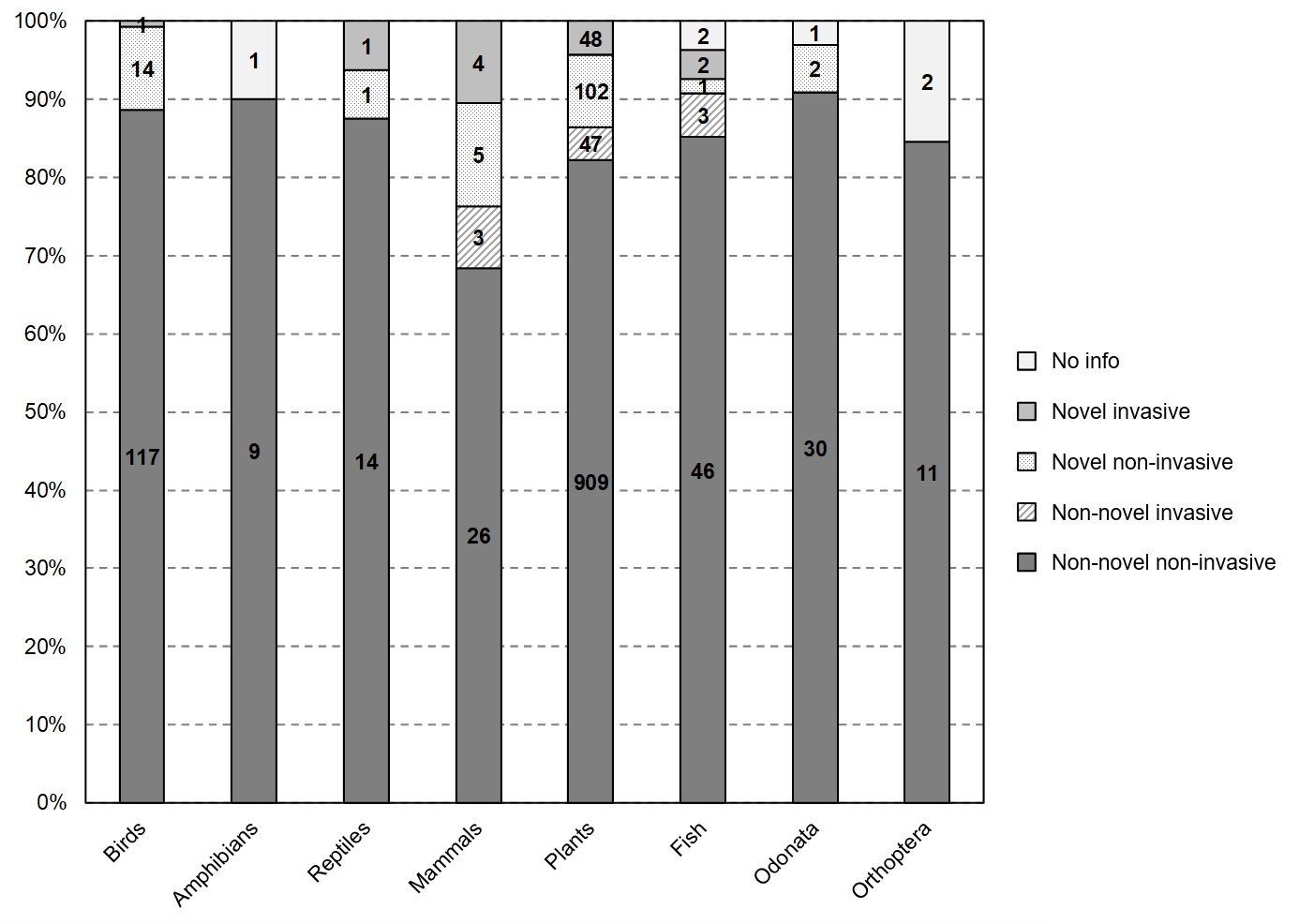

Supplement: S1 Fig — Number of species classified in each of the four categories of the variable “nov-inv”: non-novel non-invasive species, non-novel invasive species, novel non-invasive species and novel invasive species for each taxonomic group. Species for which we could not confirm whether they were considered as new arrivals are also plotted. Note that numbers have been obtained from the trend database (n = 1402 species). Categories are represented as percentages in order to be compared between taxa. The majority of species were classified as non-novel non-invasive (83% from total). Amphibians and odonates had 90% or higher number of species belonging to this category. Novel non-invasive species were the second most popular group (9% from total). Mammals had most species belonging to categories non-novel invasive (8%), novel non-invasive (13%) and novel invasive (11%). (TIF) [file pone.0224235.s010.tif]
