# Supplementary material for: Estimating biodiversity changes in the Camargue wetlands: An expert knowledge approach
Source: PLoS One. 2019 Oct 24;14(10):e0224235. doi: 10.1371/journal.pone.0224235 (PMC6812746; doi:10.1371/journal.pone.0224235)
Supplement: S2 Table — Trend estimates from experts were compared to trends derived from quantitative data, only known for species counted in all or almost the entire delta. Species with one asterisk refer to one category of discrepancy among trends (e.g. stable vs increase), whereas two asterisks indicate a two-level discrepancy among trends (i.e. opposite trends; decline vs increase). (DOCX) [file pone.0224235.s006.docx]

| **Species** | **Trend literature** | **Trend experts** |
| --- | --- | --- |
| **Birds** | | |
| *Anser anser* | ↑ | ↑ |
| *Cygnus olor* | ↑ | ↑ |
| *Phalacrocorax carbo* | ↑ | ↑ |
| *Ardea alba* | ↑ | ↑ |
| *Ardea cinerea* | ↑ | ↑ |
| *Ardea purpurea* * | ↓ | ≈ |
| *Ardeola ralloides* | ↑ | ↑ |
| *Bubulcus ibis* | ↑ | ↑ |
| *Egretta garzetta* * | ↑ | ≈ |
| *Nycticorax nycticorax* | ≈ | ≈ |
| *Ciconia ciconia* | ↑ | ↑ |
| *Platalea leucorodia* | ↑ | ↑ |
| *Plegadis falcinellus* | ↑ | ↑ |
| *Threskiornis aethiopicus* | ↑ | ↑ |
| *Pheoniconaias minor* | ↑ | ↑ |
| *Phoenicopterus roseus* | ↑ | ↑ |
| *Buteo buteo* | ↑ | ↑ |
| *Circus pygargus* | ↓ | ↓ |
| *Porphyrio porphyrio* | ↑ | ↑ |
| *Recurvirostra avosetta* | ↓ | ↓ |
| *Burhinus oedicnemus* ** | ↓ | ↑ |
| *Glareola pratincola* | ↑ | ↑ |
| *Chroicocephalus genei* | ↑ | ↑ |
| *Chroicocephalus ridibundus* | ↓ | ↓ |
| *Ichthyaetus melanocephalus* | ↑ | ↑ |
| *Larus michahellis* * | ↑ | ≈ |
| *Chlidonias hybrida* | ↓ | ↓ |
| *Gelochelidon nilotica* * | ↓ | ≈ |
| *Sterna hirundo* | ↓ | ↓ |
| *Sternula albifrons* | ↓ | ↓ |
| *Thalasseus sandvicensis* | ↓ | ↓ |
| *Columba palumbus* | ↑ | ↑ |
| *Streptopelia decaocto* | ↑ | ↑ |
| *Tyto alba* | ↓ | ↓ |
| *Athene noctua* | ↓ | ↓ |
| *Bubo bubo* | ↑ | ↑ |
| *Otus scops* | ↓ | ↓ |
| *Calandrella brachydactyla* | ↓ | ↓ |
| *Melanocorypha calandra* | ↓ | ↓ |
| *Motacilla alba* | ↑ | ↑ |
| *Troglodytes troglodytes* | ↑ | ↑ |
| *Phoenicurus ochruros* | ↑ | ↑ |
| *Turdus merula* | ↑ | ↑ |
| *Sylvia communis* | ↓ | ↓ |
| *Remiz pendulinus* | ↓ | ↓ |
| *Lanius meridionalis* | ↓ | ↓ |
| *Lanius minor* | ↓ | ↓ |
| *Corvus corone* | ↑ | ↑ |
| *Corvus frugilegus* | ↑ | ↑ |
| *Petronia petronia* | ↑ | ↑ |
| **Mammals** | | |
| *Genetta genetta* | ↑ | ↑ |
| *Martes foina* | ↑ | ↑ |
| *Martes martes* | ↑ | ↑ |
| *Eliomys quercinus* | ↓ | ↓ |
| *Myocastor coypus* * | ↑ | ≈ |
| *Ondatra zibethicus* | ↑ | ↑ |
| *Sciurus vulgaris* | ↑ | ↑ |
| *Crocidura suaveolens* | ↓ | ↓ |
| *Capreolus capreolus* | ↑ | ↑ |
| *Sus scrofa* | ↑ | ↑ |
| *Oryctolagus cuniculus* | ↓ | ↓ |
| *Lepus europaeus* | ↓ | ↓ |
| *Rhinolophus ferrumequinum* | ↓ | ↓ |
