# Supplementary material for: Estimating biodiversity changes in the Camargue wetlands: An expert knowledge approach
Source: PLoS One. 2019 Oct 24;14(10):e0224235. doi: 10.1371/journal.pone.0224235 (PMC6812746; doi:10.1371/journal.pone.0224235)
Supplement: S3 Table — Number and percentage of increasing, declining and stable species for each of the taxonomic groups evaluated based on the average reply from experts (raw trend data). Categorization of trends was the same for all taxonomic groups (see S2 Appendix)). The highest percentages of declining species were identified for orthopterans, odonates and amphibians, while the highest percentage of increasing species belonged to mammals, plants and birds. The highest percentages of stable species were associated to fish, followed by birds and plants. For half of the taxonomic groups, the percentage of stable species was a bit higher than for increasing and declining species. (DOCX) [file pone.0224235.s007.docx]

| **Taxonomic group** | **Number of species** | **Declining trend** | **Stable trend** | **Increasing trend** |
| --- | --- | --- | --- | --- |
| Birds | 132 | 29 [22%] | 63 [48%] | 40 [30%] |
| Amphibians | 10 | 8 [80%] | 0 [0%] | 2 [20%] |
| Reptiles | 16 | 8 [50%] | 5 [31%] | 3 [19%] |
| Mammals | 38 | 11 [29%] | 14 [37%] | 13 [34%] |
| Vascular plants | 1106 | 257 [23%] | 501 [45%] | 348 [31%] |
| Fish | 54 | 12 [22%] | 36 [67%] | 6 [11%] |
| Odonates | 33 | 27 [82%] | 1 [3%] | 5 [15%] |
| Orthopterans | 13 | 11 [85%] | 0 [0%] | 2 [15%] |
| **All** | **1402** | **363 [26%]** | **620 [44%]** | **419 [30%]** |
