# Supplementary material for: Estimating biodiversity changes in the Camargue wetlands: An expert knowledge approach
Source: PLoS One. 2019 Oct 24;14(10):e0224235. doi: 10.1371/journal.pone.0224235 (PMC6812746; doi:10.1371/journal.pone.0224235)
Supplement: S4 Table — Number of species distributed in the different abundance categories for each taxonomic group and period (the 1970s and the 2010s) based on the average answer from experts (raw abundance data). Categorization of abundance was the same for amphibians, reptiles, mammals, fish, odonates and orthopterans (see S2 Appendix). For birds, numbers in brackets refer to individuals. (DOCX) [file pone.0224235.s008.docx]

| **Taxonomic group** | **A**  **[Absent]** | **RL**  **[Rare, localized]** | **RW**  **[Rare, widespread]** | **CL**  **[Common, localized]** | **CW**  **[Common, widespread]** | **Total** |
| --- | --- | --- | --- | --- | --- | --- |
| Amphibians 1970 | 0 | 1 | 2 | 1 | 5 | 9^a^ |
| Amphibians 2010 | 0 | 2 | 1 | 4 | 3 | 10 |
| Reptiles 1970 | 2 | 2 | 3 | 3 | 6 | 16 |
| Reptiles 2010 | 0 | 4 | 3 | 4 | 5 | 16 |
| Mammals 1970 | 11 | 13 | 2 | 12 | 20 | 58 |
| Mammals 2010 | 1 | 23 | 9 | 5 | 20 | 58 |
| Fish 1970 | 2 | 6 | 7 | 20 | 18 | 53^a^ |
| Fish 2010 | 1 | 8 | 5 | 18 | 21 | 53 |
| Odonates 1970 | 5 | 20 | 1 | 19 | 8 | 53^b^ |
| Odonates 2010 | 3 | 17 | 12 | 11 | 12 | 55 |
| Orthopterans 1970 | 0 | 1 | 0 | 0 | 0 | 1^c^ |
| Orthopterans 2010 | 1 | 13 | 13 | 18 | 30 | 75^c^ |

^a^one species for which we do not know the abundance

^b^two species for which we do not know the abundance

^c^83 species for which we do not know the abundance (1970s) / nine species for which we do not know the abundance (2010s)

| **Taxonomic group** | **A**  **[0]** | **B**  **[1–10]** | **C**  **[10–10^2^]** | **D**  **[10^2^–10^3^]** | **E**  **[10^3^–10^4^]** | **F**  **[10^4^–10^5^]** | **Total** |
| --- | --- | --- | --- | --- | --- | --- | --- |
| Birds 1970 | 17 | 9 | 30 | 42 | 31 | 3 | 132 |
| Birds 2010 | 7 | 14 | 17 | 60 | 31 | 3 | 132 |

| **Taxonomic group** | **A**  **[Absent]** | **Vr**  **[Very rare]** | **R**  **[Rare]** | **Lf**  **[Less frequent]** | **C**  **[Common]** | **Vc**  **[Very common]** | **Total** |
| --- | --- | --- | --- | --- | --- | --- | --- |
| Plants 1970 | 148 | 366 | 243 | 263 | 117 | 22 | 1159^a^ |
| Plants 2010 | 41 | 382 | 303 | 312 | 110 | 18 | 1166^a^ |

^a^104 species for which we do not know the abundance (1970s) / 97 species for which we do not know the abundance (2010s)
