# Supplementary material for: Estimating biodiversity changes in the Camargue wetlands: An expert knowledge approach
Source: PLoS One. 2019 Oct 24;14(10):e0224235. doi: 10.1371/journal.pone.0224235 (PMC6812746; doi:10.1371/journal.pone.0224235)
Supplement: S5 Table — Average weighted species trends, abundances, distributions (except for birds and vascular plants) and confidence scores for each taxonomic group. See S2 Appendix) for more information on the different categories and given values. Note that only one orthopteran species was evaluated in the 1970s. (DOCX) [file pone.0224235.s009.docx]

| **Taxonomic group** | **Trend** | **CS** | **Ab**  **1970** | **Dist**  **1970** | **CS**  **1970** | **Ab**  **2010** | **Dist**  **2010** | **CS**  **2010** |
| --- | --- | --- | --- | --- | --- | --- | --- | --- |
| Birds | −0.02 | 0.34 | 2.44 | − | 0.18 | 2.74 | − | 0.29 |
| Vascular plants | 0.08 | 0.15 | 1.91 | − | 0.17 | 2.10 | − | 0.19 |
| Amphibians | −0.55 | 0.51 | 1.64 | 1.57 | 0.46 | 1.68 | 1.35 | 0.60 |
| Reptiles | −0.28 | 0.51 | 1.50 | 1.27 | 0.51 | 1.56 | 1.41 | 0.61 |
| Mammals | −0.03 | 0.37 | 1.33 | 1.23 | 0.27 | 1.39 | 1.36 | 0.37 |
| Fish | −0.14 | 0.50 | 1.56 | 1.46 | 0.49 | 1.65 | 1.52 | 0.63 |
| Odonates | −0.67 | 0.33 | 1.42 | 1.08 | 0.25 | 1.40 | 1.15 | 0.82 |
| Orthopterans | −0.69 | 0.15 | − | − | − | 1.56 | 1.54 | 0.42 |
